# Immune checkpoint inhibitor-induced pure red cell aplasia: a nationwide retrospective case series and literature review

**DOI:** 10.1007/s00277-026-06748-0

**Published:** 2026-01-20

**Authors:** Sirivanh Bisiou, Hervé Lobbes, Pascale Palassin, Marion Allouchery, Thibault Comont, Valérian Rivet

**Affiliations:** 1 Department of Medecine and Clinical Immunology, Montpellier University Hospital, Montpellier, France; 2 Internal Medicine Department, Clermont Ferrand University Hospital, Clermont-Ferrand, France; 3 Department of Medical Pharmacology and Toxicology, Regional Pharmacovigilance Center, CHRU Montpellier, Montpellier, France; 4 Clinical Pharmacology and Vigilance Department, Poitiers University Hospital, Poitiers, France; 5 Department of Internal Medicine and Immunopathology, Institut du Cancer de Toulouse, Toulouse, France; 6 Department of Internal Medicine and Immunopathology, Toulouse University Hospital, Toulouse, France

## Abstract

Immune checkpoint inhibitors (ICIs) may lead to rare but severe hematological adverse events, including pure red cell aplasia (ICI-PRCA). We conducted a nationwide retrospective national case-series and compiled our data with patients from the VigiBase international pharmacovigilance database. We gathered 16 cases of ICI-PRCA Among all the grade ≥ 3 adverse event reported in VigiBase, PRCA was more than two times more reported with ICI compared to all other drugs ROR [IC95%] 2.43 [1.89;3.13]. We identified 15 additional patients in our literature review. Half of the patients were women (52%), median age 63 years (range: 29–90). ICIs were mostly used in melanoma (48%) and lung adenocarcinoma (16%) in metastatic stage (29%). Monoclonal antibody targeting programmed death-1 (PD-1) alone was involved in 61% of cases. The median time from ICI initiation to symptoms onset was 63 days (range: 28–465). Grade ≥3 anemia in found in all cases (100%). Thirteen patients (42%) were treated with corticosteroids as monotherapy with an ORR of 69%. Thirteen patients (42%) required additional therapy. ICI therapy was discontinued in all patients and rechallenge was attempted in 4 patients (13%), with recurrence in one case. ICI-PRCA is a severe and early-onset immune adverse event. Both systemic steroids and cicloporine A seems effective in these immune-related forms.

 Immune checkpoint inhibitors (ICIs) may lead to rare but severe hematological adverse events (irAES-hem), occurring in approximately 3.6% of patients [1, 2]. Pure red cell aplasia (PRCA) induced by ICIs (ICI-PRCA) are scarcely reported and may be life threatening [1, 2]. We conducted a nationwide retrospective national case-series and compiled our data with patients from the VigiBase international pharmacovigilance database including.

Adult’s patients (≥ 18 years) who received ICI and have been diagnosed with PRCA, up to July 2023 were included. All others causes of PRCA were excluded. The study was conducted in accordance with the declaration of Helsinki and approved by the local Ethics Committee (IRB00013412, “CHU de Clermont-Ferrand IRB #1”, IRB number 2022-CF040) in compliance with French data protection regulations. Cases from the VigiBase database were selected through individual case safety report (ICSR) of “erythroblastopenia” reported with all ICI using the narrow Standardized Drug Groupings (SDG) ‘Antineoplastic immune checkpoint inhibitors’. All extracted ICSR are from France including eight from the French Pharmacovigilance Database (Fig. 1.). Disproportionality analyses were conducted using the case/non-case method which allows to identify a disproportionate reporting by calculating Reporting Odds Ratios (ROR) [3]. Cases were all reports of severe PRCA and non-cases were all other serious drug-related adverse reactions recorded in this global database. Exposure to ICI was compared between cases and non-cases to all other drugs and, in a secondary analysis, to all other cancer therapies. A signal of disproportionate reporting was considered if the 95% confidence interval (95% CI) lower limit of the ROR exceeded 1. Complete response (CR) was defined as normalization of hemoglobin levels 8 weeks after treatment, allowing for transfusion independence. Partial response (PR) was characterized by an increase in reticulocyte count, leading to partial correction of hemoglobin levels. Non-response (NR) was defined by the absence of both CR and PR criteria [4].

**Fig. 1:**
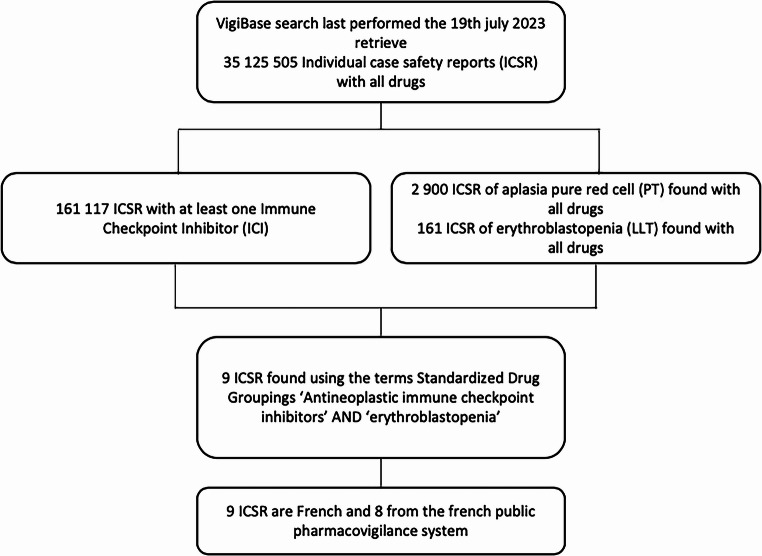
Flow chart ofpharmacovigilance data extraction in the global database, VigiBase Among 35,125,505 adverse drug reactions in the VigiBase database, 161,117 cases were linked to ICI

We gathered 16 cases of ICI-PRCA: 3 patients treated at the Toulouse University Hospital, 5 patients from the French PRCA National cohort, 8 patients from the French pharmacovigilance database. Among all the grade ≥ 3 adverse event reported in the international Pharmacovigilance database, PRCA was more than two times more reported with ICI compared to all other drugs and other cancer therapies: ROR [IC95%] 2.43 [1.89;3.13] and 2.56 [1.96;3.35] respectively. We identified 15 additional patients in our literature review. Half of the patients were women (52%), median age 63 years (range: 29–90) (Table 1.). ICIs were mostly used in melanoma (48%) and lung adenocarcinoma (16%) in metastatic stage (29%). Monoclonal antibody targeting programmed death-1 (PD-1) alone was involved in 61% of cases. The median time from ICI initiation to symptoms onset was 63 days (range: 28 to 465 days), therefore, mostly within the first 3 months of treatment, defined as early-onset toxicity [5]. Twelve patients (44%) experienced at least one other irAE in the same time or before. Laboratory tests revealed CTCAE (v 5.0) grade ≥3 anemia in all cases (100%) (mean nadir hemoglobin 6.2 g/dl) [6]. Packed red blood cell transfusion was required in 22 cases (71%). Bone marrow examination showed absence of erythroid precursors for all cases: detailed results were available for 10 patients (32%), showing normal cellularity and absence of blast cells or neoplastic infiltrates. A polyclonal T-CD8 + lymphocytic infiltrate was observed in all cases.

**Table 1:** Main characteristics of patients with immune-related pure red cell aplasia of previously published, current cases and pharmacovigilance database patients

Characteristic	IUCT-EPICF-PV (*n* = 16)		Literature review (*n* = 15)	Total (*n* = 31)
Sex ratio female / male	0,8		1,8	1,1
Median age (range min-max)	63 (41–90)		60 (29–75)	63 (25–90)
Dysimmune medical history (n)	5		NR	5
**Time to onset**,** median number of cycles (days)**	4 (63)		4 (80)	4 (63)
**ICI regimens**,** n (%)**				
Nivolumab (%)	7 (44)		3 (20)	10 (33)
Pembrolizumab (%)	5 (32)		4 (27)	9 (29)
Atezolizumab (%)	2 (12)		1 (6,5)	3 (10)
Durvalumab (%)	0 (0)		2 (13)	2 (6)
Ipilimumab (%)	0 (0)		1 (6,5)	1 (3)
ICI combination (nivolumab + ipilimumab)	2 (12)		4 (27)	6 (20)
**Therapeutic indication**,** n (%)**				
Melanoma	7 (44)		8 (53)	15 (48)
Lung carcinoma	3 (19)		2 (12)	5 (16)
Breast cancer	1 (6)		1 (7)	2 (6)
Mesothelioma	2 (12)		0 (0)	2 (6)
Renal neoplasia	2 (12)		0 (0)	2 (6)
Sarcoma	0 (0)		1 (7)	1 (3)
Lymphoma	0 (0)		1 (7)	1 (3)
Prostatic cancer	0 (0)		1 (7)	1 (3)
NC (%)	1 (6)		1 (7)	2 (6)
**TNM oncologic stage**,** n (%)**				
I/II	2 (12)		0 (0)	2 (6)
III	3 (19)		4 (27)	7 (23)
IV	10 (63)		11 (73)	21 (68)
NC	1 (6)		0 (0)	1 (3)
**ICI-associated irAES**,** n (%)**				
AHAI	2 (12)	0 (0)		2 (6)
Thrombopenia	1 (6)	0 (0)		1 (3)
Neutropenia	1 (6)	0 (0)		1 (3)
Colitis	1 (6)	1 (7)		2 (6)
Hepatitis	2 (12)	1 (7)		3 (10)
Cutaneous (Vitiligo)	0 (0)	2 (12)		2 (6)
Dysthyroidism	0 (0)	3 (20)		3 (10)
Demyelinating disease	0 (0)	1 (7)		1 (3)
Tubulointerstitial nephritis	1 (6)	0 (0)		1 (3)
**Biological and histological features**				
Hb, mean - g/dl	8,0		6,9	7,5
Nadir Hb, mean - g/dl	6,4		5,8	6,2
Reticulocytes count, Mean - g/dl	8,8		2,6	6,4
CTCAE grade*	3		3	3
Lymphocytic CD8+ infiltration in BME (%)	5/5 (100)		5/5 (100)	10/10 (100)

*IUCT* Institut Universitaire du Cancer de Toulouse, *EPICF* Erythroblastopénies auto-immunes, cohorte nationale Française, *PV* Pharmacovigilance database, *ICI* Immune-checkpoint inhibitor, *anti*-*PD1* antibody targeting programmed death-1, *PD*-*L1* programmed cell death ligand 1, *CTLA*-*4* cytotoxic T lymphocyte associated antigen-4, *NC* not communicated, *CTCAE* *grade* Common terminology criteria for adverse events (version 5.0), *BME* Bone marrow examination, *AHAI* Auto-immune hemolytic anemia

* According to CTCAE,* version 5.0* [4]

Thirteen patients (42%) were treated with corticosteroids (Cs) as monotherapy with an ORR of 69% (5 CR [38%] ; 4 PR [13%] ; 4 NR [13%]). Thirteen patients (42%) required additional therapy: cyclosporine A (CsA) (*n* = 7, 5 CR [71%], 2 NR [29%]), intravenous immunoglobulin (IVIg) (*n* = 6, 6 CR). One patient was treated with cyclophosphamide (CYC) followed by IVIg. In all cohort, ORR was observed in 21 patients (68%) (17 CR [55%] ; 4 PR [13%] ; 10 NR [32%]). ICI therapy was discontinued in all patients and rechallenge was attempted in 4 patients (13%), with recurrence in one case.

ICI-PRCA appear in our cohort with a balanced sex ratio and age quite similar to the usual epidemiology of primary PRCA [4, 7]. As observed with other irAEs-hem, anti-PD1 and anti-PD-L1 were the most frequently used ICIs, with predominance of anti-PD1 in nearly two-thirds of the patients in our cohort [1, 2]. Like other irAEs-hem, PRCA manifests early, within the first 3 months [1, 2]. The T-CD8 + lymphocytic infiltrate found in one-third of our patients may be a significant indicator for confirming immune etiology [1].

As previously suggested by Guo et al., response to Cs seems higher in ICI-PRCA compared primary PRCA (ORR 47%), with a complete response rate of 38% (ORR 69%) in our study and 58% in Guo et al. [6, 8]. Indeed, immunosuppressive agents are often recommended in first line treatment for primitive PRCA [4, 7]. The most common first-line treatment for primary PRCA is CsA, which has an overall response rate of 71% although CYC may probably also be used. The response to IVIg is uncertain in primary PRCA (22%), while it is a recommended treatment in parvovirus B19 induced PRCA (100%) [4]. In refractory cases, CsA is effective therapy. In Guo et al., five patients who did not respond to steroids fully recovered under treatment with those therapies [8]. Further studies are still necessary to confirm optimal management strategies. 

ICI-induced PRCA is a severe and early-onset immune adverse event. Both systemic corticosteroid and ciclosporine A appear to be effective treatments, as observed in the primary forms, leading to resolution of anemia in the majority of patients. Data are still missing to discuss rechallenge after this toxicity.

## Data Availability

The original contributions presented in the study are included in the article/supplementary material. Further inquiries can be directed to the corresponding authors.
